# Screening four broad categories of eating disorders: suitability of a clinical algorithm adapted from the SCOFF questionnaire

**DOI:** 10.1186/s12888-019-2338-6

**Published:** 2019-11-21

**Authors:** Marie-Pierre Tavolacci, André Gillibert, Aurélien Zhu Soubise, Sébastien Grigioni, Pierre Déchelotte

**Affiliations:** 1 Normandie Univ, UNIROUEN, U1073, Rouen University Hospital, Clinical Investigation Center 1404, F-76000 Rouen, France; 2 Rouen University Hospital, Clinical Investigation Center 1404, F-76000 Rouen, France; 3 Department of Nutrition, Normandie Univ, UNIROUEN, U1073, Rouen University Hospital, F-76000 Rouen, France

**Keywords:** Screening, Algorithm, Eating disorders, General practice, SCOFF questionnaire

## Abstract

**Background:**

We evaluated the performance of a clinical algorithm (Expali**™),** combining two or more positive answers to SCOFF questionnaire with Body Mass Index (BMI), to identify four Broad Categories of eating disorders (ED) derived from DSM-5.

**Methods:**

The clinical algorithm (Expali**™)** was developed from 104 combinations of BMI levels and answers to five SCOFF questions with at least two positive answers. Two senior ED physicians allocated each combination to one of the four Broad Categories of ED derived from DSM-5: restrictive disorder, bulimic disorder, hyperphagic disorder and other unspecified ED diagnosed by ED clinicians. The performance of Expali**™** was evaluated on data from 206 patients with ED. Sensitivity, specificity values and Youden index were calculated for each category.

**Results:**

The 206 patients were diagnosed as follows: 31.5% restrictive disorder, 18.9% bulimic disorder, 40.8% hyperphagic disorder and 8.8% other ED. The sensitivity of Expali**™** for restrictive, bulimic, hyperphagic and other unspecified ED were respectively: 76.9, 69.2, 79.7 and 16.7%. The Youden index was respectively 0.73, 0.57, 0.67 and 0.07.

**Conclusions:**

In a SCOFF-positive ED population (at least two positive answers), the clinical algorithm Expali**™** demonstrated good suitability by correctly classifying three of the four Broad Categories of eating disorders (restrictive, bulimic and hyperphagic disorder). It could be useful both to healthcare professionals and the general population to enable earlier detection and treatment of ED and to improve patient outcomes.

## Background

The Diagnostic and Statistical Manual of Mental Disorders-version 5 (DSM-5), distinguishes eating disorders (ED) into six main diagnostic categories: Restrictive food intake disorder, anorexia nervosa (AN), bulimia nervosa (BN), binge eating disorder (BED), rumination disorder, and a residual category renamed Other Specified Feeding and Eating Disorders (OSFED) (annex 1) [1]. For the typical ED (AN, BN), data on course and outcome are not significantly influenced by changes in definition between the current DSM-5 and the former DSM-IV, a finding consistent with earlier observations that the outcome of atypical (subsyndromic) AN or BN was quite similar to that of typical AN or BN in DSM-IV [2].

Based on DSM-5, the lifetime prevalence of eating disorders ranges from 9 to12% [2–4]. A recent literature review indicated that BED then BN and AN had the highest lifetime prevalences [5]. Despite this high prevalence, ED are often poorly detected in the general population, resulting in delayed treatment [6]. Early detection and treatment of ED lead to better outcomes [7, 8] since ED are associated with considerable physical and psychiatric comorbidities [9].

The early detection and treatment of ED is of key importance because of the life-threatening complications of BN and AN [10]. Unfortunately, most GPs do not feel comfortable with the screening of ED. This is often associated with delayed diagnosis especially during primary care consultation [11, 12]. Indeed, fewer than 10% of cases of BN and BED and fewer than 50% of cases of AN and subclinical AN are detected by GPs [13]. Although there is improved awareness of ED as a whole among clinicians, there is a lack of understanding of BED as a distinct ED, which may result in low rates of screening and diagnosis of this most frequent ED [14]. Therefore, in order to simplify the diagnostic approach in primary care, broader diagnostic categories of ED have been proposed by some authors [15, 16]. AN and behaviorally similar disorders, BN and behaviorally similar disorders, and BED and behaviorally similar disorders; as in DSM-IV and DSM-5, the broad categories also include a residual Eating Disorders Not Otherwise Specified (EDNOS) category [17].

To facilitate the screening of ED in primary care, the SCOFF-test (Sick, Control, One stone, Fat, Food) has been developed [18]. At least 2 positive answers indicate a positive SCOFF with a good sensitivity (88.2%) and specificity (92.5%) [19]. The NICE guidelines suggest using the SCOFF test as a screening tool for ED in primary care [20].

AN and BN share common features such as perfectionism, obsessive-compulsiveness, neuroticism, and negative emotionality, which may not be easy to detect by non-specialists [21]. In contrast, a self-perception of being too fat, or fear of gaining weight is present in both AN and BN and both are easily expressed by patients [22]. High impulsivity, sensation seeking, and loss of control are typical of bulimic disorders in, such as BN or BED but BMI is usually normal in BN while it is increased in BED [23]. Purging behaviors, mainly vomiting or laxative abuse, are found both in AN- and BN-purging type, but BMI is decreased only in the former. Recent and significant weight loss is highly indicative of an ongoing restrictive disorder, with or without purging. Although loss of control (binging) may be undeclared by patients, and underdiagnosed by physicians, impulsive/compulsive behaviors should be screened in patients referred for weight excess and or recent weight gain [24]. BMI alone does not allow the detection of a specific type of ED, as not all obese patients have an active ED, patients with BED may have normal BMI because BED alternates with restrictive periods and patients with BN are not obese [25]. In addition, low BMI may be related to other causes of malnutrition than restrictive ED including AN.

Thus, a simple tool combining easily and quickly available data such as BMI and individual answers to the SCOFF test could be very useful to support diagnosis of ED in primary care at the same time as screening. With this in mind, we developed a clinical algorithm (Expali**™**) combining two or more positive answers to SCOFF and four levels of BMI and evaluated its ability to identify four broad categories of ED derived from DSM-5.

## Methods

### Setting

This observational study included patients referred to the adult Nutrition Department of Rouen University Hospital for ED in November and December 2015. The study was approved by Rouen University Hospital’s Institutional Review Board (E2017–10) without written consent because of non-interventional research and has been registered in ClinicalTrials.gov ID: NCT03208699.

### Participants

All consecutive patients aged 18 and over were included whatever the comorbidities (somatic or mental disorders. For each patient, the specialist physician diagnosed the precise type of ED according to DSM-5. To avoid the own biases of the clinicians, each diagnosis was confirmed by the senior ED physician.

### Data collection

#### Body mass index

Patients were classified into four categories based on their body mass index (BMI): underweight (BMI < 18.5), normal (BMI between 18.5 and 24.9), overweight (BMI between 25.0 and 29.9), and obese (BMI > 30) [26].

#### SCOFF test

A positive SCOFF score indicates an ED with at least 2 positive answers to the 5 questions [18]. The questionnaire has been translated into French (SCOFF-F) ([27]**Error! Bookmark not defined.**). Patients referred for a nutritional disorder but not diagnosed as having an ED and with a SCOFF score equal to 0 or 1 were not included.

The five SCOFF questions are binary coded (Yes/No) and the original ordering is as follows: *Do you make yourself* ***Sick*** *because you feel uncomfortably full? Do you worry that you have lost* ***Control*** *over how much you eat? Have you recently lost more than* ***One*** *stone (6.35 kg) in a 3-month period? Do you believe yourself to be* ***Fat*** *when others say you are too thin? Would you say that* ***Food*** *dominates your life?*. Patients with at least two positive answers to the SCOFF test and fulfilling study criteria were included.

#### Classification of four broad eating disorders derived from DSM-5

Four Broad Categories for the Diagnosis of Eating Disorders, similar to those previously reported for DSM-IV [15], were created based on the DMS-5 categories of ED (Annex A): **1- Restrictive disorders (RD)** including anorexia nervosa, restrictive food intake disorder, and atypical anorexia nervosa; **2- Bulimic disorders (BD)** including bulimia nervosa or bulimia nervosa of low frequency or duration; **3- Hyperphagic disorders (HD)** including Binge Eating Disorder and Binge Eating Disorder of low frequency or duration; and **4: Other eating disorders (OED)** including Purging disorder, and Night Eating Syndrome and any other ED.

#### The clinical algorithm: Expali**™**

We have developed a clinical algorithm combining at least two positive answers to the SCOFF test and one of the four BMI levels. Expali™ has a total of 104 possible combinations of SCOFF answers and BMI levels as only SCOFF ≥2 were included in the algorithm. Two senior ED physicians individually assigned each of the 104 combinations to one of the four broad categories of ED (RD, BD, HD or OED) We had planned a third competent judge if there was discrepancy between the two experts, however they agreed for all combinations. As a result, 29 combinations were assigned to RD, 34 to BD, 15 to HD and 26 to OED (Table 1). For example, the combination of positive answers to SCOFF items 3 and 4 (weight loss and feeling fat) and a BMI of 16.5 were assigned to RD; the combination of positive answers to SCOFF items 1 and 2 (sick, loss of control) and a BMI of 21 were assigned to BD; the combination of positive answers to SCOFF items 2 and 5 (loss of control, food dominates) and a BMI of 34 were assigned to HD. A copyright of the algorithm has been registered under the name of Expali™ (EXPert system ALImentary) by Rouen University Hospital.

**Table 1:** Number of possible combinations of answers to SCOFF test and BMI level in the clinical algorithm Expali**™**

	Restrictive Disorders (RD)	Bulimic Disorders (BD)	Hyperphagic Disorders (HD)	Other Eating Disorders (OED)	Total combinations of SCOFF answers and BMI levels
Underweight	26	0	0	0	26
Normal	3	18	1	4	26
Overweight	0	8	7	11	26
Obese	0	8	7	11	26
Total combinations	29	34	15	26	104

### Statistics

#### Validation of Expali**™**

The validity of the clinical algorithm (Expali**™**) was assessed by calculating sensitivity and specificity values for each of the four broad categories (RD, BD, HD and OED). The gold standard was the senior ED physician’s diagnosis of the patient’s ED according to DSM-5. For each diagnosis of ED, the group of true negatives was the sum of patients with the 3 other broad categories. Sensitivity is defined as the percentage of patients with the category of ED correctly identified by Expali**™** i.e. the same category of ED that assigned by the senior ED physician (gold standard). Specificity is defined as the percentage of patients correctly identified by Expali**™** as not having the specific diagnosis under investigation. Youden’s Index was calculated by the following equation: sensitivity+ specificity − 1. The Youden index score ranges from 0 to 1 with a score of 1 indicating that Expali**™** has perfect diagnostic performance for a specific diagnosis category and a score close to 0 indicating the same performance as random classification. The Youden index was calculated for each diagnosis category. Cohen’s kappa coefficient was calculated to assess the global concordance between Expali**™** and the diagnosis given by the ED physicians with a 95% Confidence Interval (CI). There were no missing data.

## Results

### Characteristics of patients

In November and December 2015, 259 patients fulfilled the diagnostic criteria for a DSM-5 ED and filled the SCOFF-F questionnaire. Among these 259 patients, 216 had a positive SCOFF- score (2 or more positive answers) and 206 of them also had BMI measurement. The precise DMS-5 diagnoses of the 206 patients are displayed in Table 2. These DSM-5 diagnoses were aggregated in each of the 4 Broad Categories as 65 RD (31.5%), 39 BD (18.9%), 84 HD (40.8%) and 18 OED (8.8%). Among these 206 SCOFF-positive patients, 199 (95.6%) were female and the mean age was 36.1 years (Standard deviation: 14.4). Patients’ characteristics according to ED diagnosis are presented in Table 3. Regarding BMI for each broad category of ED: 78.5% of patients with RD were classified as underweight, 48.7 and 30.7% of patients with BD were classified as having normal BMI and as obese respectively, 95.2% of patients with HD were classified as obese, 39.3% with grade 3 obesity, and as expected, the values of patients in the residual OED category were quite scattered.

**Table 2:** Categorisation of the 206 patients in the four broad categories of ED according the DSM-5 eating disorders classification

	Broad Categories of ED in Expali™				
	Restrictive Disorders	Bulimic Disorders	Hyperphagic Disorders	Other Eating Disorders	Total
Eating disorders according to DSM-5					
Anorexia Nervosa	39				39
Bulimia Nervosa		28			28
Binge Eating Disorder			26		26
Restrictive Food Intake Disorder	16				16
Atypical Anorexia Nervosa	10				10
Bulimia Nervosa (of low frequency and/or limited duration):		11			11
Binge Eating Disorder (of low frequency and/or limited duration):			58		58
Purging Disorder				0	0
Night Eating Syndrome				3	3
Any other eating disorders				15	15
Total	65	39	84	18	206

**Table 3:** Sex, age and BMI of the 206 patients according to the four broad Eating Disorders categories based on physicians diagnosis

	Restrictive Disorders (*n* = 65)	Bulimic Disorders (*n* = 39)	Hyperphagic Disorders (*n* = 84)	Other Eating Disorders (*n* = 18)	p	Total (*n* = 206)
Female %	98.4	100	91.7	94.4	0.10	95.6
Age mean (SD)	30.4 (12.1)	28.5 (9.8)	43.9 (13.9)	37.1 (15.6)	0.017	36.1 (14.4)
BMI Mean (SD)	17.6 (3.8)	25.9 (8.1)	39.1 (7.0)	30.8 (8.3)	< 10^−4^	29.1 (11.3)
BMI Class %					< 10^−4^	
Underweight	78.5	10.2	0	5.6		27.2
Normal	18.5	48.7	2.4	22.2		17.9
Overweight	1.5	10.2	2.4	11.1		4.4
Obesity	1.5	30.7	95.2	61.1		50.5

*OED* Other Eating Disorders, *BMI* Body Mas Index, *SD* Standard Deviation

### Results of the clinical algorithm: Expali**™**

Expali™ detected 50/65 RD, 27/39 BD, 67/84 HD and 3/18 OED (Table 4).. Sensitivity, specificity and Youden index to identify the four broad categories of ED are presented in Table 5. In this sample, the highest sensitivity was for the diagnosis of HD: 79.7%, (95% CI; 70.6–88.9) and the highest specificity for the diagnosis of RD: 96.4%, (95% CI; 93.0–99.9). The Youden indexes of Expali**™** to identify ED categories were respectively 0.73 for RD, 0.57 for BD, 0.67 for HD and 0.07 for OED. The Cohen kappa coefficient of Expali**™** with physician’s diagnosis was 0.59, (95%CI; 0.51–67).

**Table 4:** Agreement between the four broad categories of ED indentified by Expali**™** and by physicians among the 206 patients

	Expali™ category				
Physicians diagnosis	Restrictive Disorders	Bulimic Disorders	Hyperphagic Disorders	Other Eating Disorders	Total physicians diagnosis
Restrictive Disorders	50	6	3	6	65
Bulimic Disorders	4	27	7	1	39
Hyperphagic Disorders	0	6	67	11	84
Other Eating Disorders	1	9	5	3	18
Total Expali**™** information	55	48	82	21	206

**Table 5:** Sensitivity, specificity and Younden Index of the clinical algorithm Expali**™** according to the Eating Disorders category

	Restrictive Disorders (*n* = 65)	Bulimic Disorders (*n* = 39)	Hyperphagic Disorders (*n* = 84)	Other Eating Disorders (*n* = 18)
Sensitivity n - %	50/65–76.9%	27/39–69.2%	67/84–79.7%	3/18–16.7%
(95% CI)	(65.9–87.9)	(53.5–85.0)	(70.6–88.9)	(0.0–36.7)
Specificity n - %	136/141–96.4%	146/167–87.4%	107/122–87.7%	170/188–90.4%
(95% CI)	(93.0–99.9)	(82.1–92.8)	(81.5–93.9)	(86.0–94.9)
Youden Index	0.73	0.57	0.67	0.07

*CI* Confidence Interv

## Discussion

In the present study, we used Broad Categories for the Diagnosis of Eating Disorders in a way similar to that previously described [16, 17]. By doing this, we simply pooled the typical ED and related atypical or low-frequency disorder in the same category of DSM-5 (restrictive, bulimic, hyperphagic, residual category referred as “others”). In a SCOFF-positive ED population (at least two positive answers), the clinical algorithm Expali**™** demonstrated good performance by correctly classifying three of the four Broad Categories of ED (RD, BD and HD), with a Youden index of 0.73, 0.57 and 0.67, respectively. Since the validity of SCOFF for ED screening in a non-clinical population is already strongly established [19], the implementation of this simple and quick (2 to 3 min to fill out the form) algorithm in primary care settings and non-clinical populations could provide helpful and reliable data to identify three of the four categories of ED. However, in this study, Expali**™** did not adequately identify the residual category of OED (Youden Index of 0.07), these patients with OED being classified as having BN or BED. This is probably related to the unclear frontier between OED and low frequency bulimia or BED.

Patients with ED often present emotional difficulties and some form of denial that may prevent them seeking specific treatment from specialized health care systems [24, 28]. Primary healthcare professionals, including General Practitioners (GPs), could play a key role in the early detection of ED as they are more accessible to patients, however their knowledge of ED, namely screening and even more diagnosis, is limited. Patients with ED often refer to their GP for a variety of symptoms that may be misleading such as functional digestive disorders, fatigue, endocrine or anxiety disorders which may prevent the GP from detecting the underlying ED [29]. Awareness of a possible ED should be increased, especially in adolescents and young adults of both sexes, when a preoccupation about weight, shape or dietary intake is ever present and sometimes hidden by patients [30, 31] [32].. Early detection of ED in primary care, if possible in a sub-syndromic form, before diagnosis of ED seems to improve outcome [8]. As first-line healthcare providers, GPs could play a key role in detecting ED and coordinating care, including the management of complications and referral for specialized care and even hospitalization when needed [33].

Since obesity is rapidly growing in most countries, it is particularly important for GPs to detect HD or BD in their obese patients, so that an adequate therapeutic strategy may be set up. Obese patients with ED perceived that GPs focused more on physical ailments, were judgmental about weight, and were unable to distinguish the causative BED from the resulting obesity [34]. GPs who treat patients with ED often have negative stereotypes about obese patients, and may feel uncomfortable caring for obese patients [35]. Patients with other ED such as AN or BN also often experience a lack of understanding and empathy from untrained caregivers, which may reinforce their mistrust of the healthcare system. Stigma-reduction efforts are therefore needed in the ED field, to limit bias in the detection and treatment of ED, even among mental healthcare professionals [36].

Young adults and adolescents are particularly exposed to ED with a prevalence ranging from 14 to 20% in university populations [37–39] and 80% of university students with clinically significant symptoms do not receive care [37]. The high prevalence of mental health issues among university students unfortunately precludes providing one-on-one treatment services to all students in need of care [40]. In addition, stigma, social stereotyping and also lack of time, lack of perceived need, and a desire to deal with the issue “on my own” have been identified by university students as impediments to care for ED-related symptoms [41]. Integrating large-scale systematic detection of ED by physicians and nurses in university departments of preventive medicine could be done easily and quickly using the Expali**™** algorithm. Interventions on ED and healthy weight management in university prevention programmes could reduce the incidence and prevalence of ED, unhealthy weight control practices and later obesity among university students [42, 43].

The need for treatment of ED remains largely unaddressed by the general population, with as few as 17–31% of individuals in the community with a diagnosable ED seeking ED-specific treatment [44]. Thus, an online approach including a first-step diagnosis with Expali**™** may help patients to be better aware of their condition and may be followed by some Internet-based dynamic, evolving source of health information validated by healthcare professionals [45, 46].] Users receive individualized feedback about their clinical profile and screening results with information on specific interventions on offer; this kind of online universal or targeted preventive intervention could even be strengthened by adding a web-assisted diagnostic approach as enabled by Expali**™**.

### Strengths and limitations

One of the strengths of our study is the size of the sample with more than 200 patients with a precise diagnosis of ED according to DSM-5. The setting in a nutrition department, which is a regional reference center for ED, enabled fast recruitment. The sample included is representative of the distribution of ED: two out of five patients with BED, one out of three with AN, one out of five with BN and other patients with OED as described [3]. One limitation is the heterogeneity of patients with OED in this study, as expected for a residual category. However, the most important element in a first-step screening and diagnosis strategy, is the initial detection of an ED and thereafter further assessment for more precise diagnosis. Finally, the concept of Broad Categories was successful in reducing the number of EDs classified as OED. The precise classification of Expali**™**, for instance between typical or atypical AN in the RD category, might not be critical in a primary care setting, since the practical implications for clinical evaluation and specialized referral are quite the same, and outcome also similar. Thus, it seems important in primary care to rely on a simple tool that may help by allocating patients to a diagnosis category instead of leaving too many patients as “undefined” or “others”.

## Conclusions

Standard SCOFF with at least two “YES” answers just tells the primary health care professional that an ED is much likely present. The Expali**™** tool does not identify additional cases to standard SCOFF but allow to specify a likely diagnostic category of “restrictve”, “bulimic” or “hyperphagic” ED with only adding the BMI to the answers SCOFF. This, it will help the doctor or nurse to detect ED, even with incomplete disclosure of symptoms or normal or high BMI, and help to set up the first line of additional evaluation (e.g. checking blood potassium if bulimia is detected). Our intention is to make Expali™ freely available, but also to GPs and other primary healthcare professionals in contact with at-risk populations (adolescents, university students, patients in gastroenterology, gynecology, endocrine, and psychiatry departments), with some adapted educational content to enable formal diagnosis. The widespread use of this algorithm could also facilitate large epidemiological surveys. This validation study provides several avenues for further exploration and research. The internal validity of Expali**™** should now be assessed in a general population (in a GP or university preventive medicine setting) with precise first-step detection of ED performed by ED physicians blinded to the result of the algorithm.

## Data Availability

The dataset supporting the conclusions of this article is available in the zenodo repository: 10.5281/zenodo.1295960

## Abbreviations

AN: Anorexia Nervosa
BED: Binge eating Disorders
BMI: Body Mass Index
BN: Bulimia Nervosa
DSM: Diagnostic and Statistical Manual
ED: Eating Disorders
GP: General Practitioner
HD: Hyperphagic Disorders
OED: Other Eating Disorders
RD: Restrictive Disorders
SCOFF: Sick Control One Fat Food
